# Comments on: "Relationship involving sexual function, distress symptoms of pelvic floor dysfunction, and female genital self-image"

**DOI:** 10.61622/rbgo/2025rbgo83

**Published:** 2025-10-21

**Authors:** Hinpetch Daungsupawong, Viroj Wiwanitkit

**Affiliations:** 1 Private Academic Consultant Phonhong Lao People's Democratic Republic Private Academic Consultant, Phonhong, Lao People's Democratic Republic.; 2 Saveetha University Saveetha Institute of Medical and Technical Sciences Department of Research Analytics Chennai India Department of Research Analytics, Saveetha Dental College and Hospitals, Saveetha Institute of Medical and Technical Sciences, Saveetha University, Chennai, India.

Dear Editor,

The publication on "Relationship Involving Sexual Function, Distress Symptoms of Pelvic Floor Dysfunction, and Female Genital Self-Image"^(1)^ is hereby discussed. Although this study's stated goal was to investigate the association between GSI, SF, and PFD utilizing validated questionnaires (FGSIS, FSFI, and PFDI-20), it does have some methodological flaws that should be addressed. For example, this study employed multiple linear regression analysis, which is appropriate for investigating associations but does not reflect causality. The corrected R² values (0.097, 0.126, and 0.162) suggest that the model only explains a small portion of the dependent variable's variance, lowering confidence in the results’ interpretation.

Although the sample size was deemed enough for quantitative reasons (n=216), when only 114 people were included in the group who had sex within the previous four weeks, the analysis in this group was prone to selection bias, reducing the reproducibility of the results to the broader population. Furthermore, no controls or adjustments were made for crucial confounding factors including menopause, chronic illness, or mental health, all of which could have a considerable impact on both SF and GSI.

Revisiting this interpretation, the results demonstrate that symptoms associated with uterine prolapse (POP) are associated with GSI in both women generally and those who are sexually active. However, the relatively weak statistical relationship may indicate that GSI is impacted by a variety of social and psychological factors not examined in the study, including cultural values, knowledge of genitals, and personal ideas about beauty or femininity. The link between sexual desire and GSI may suggest that "feeling good about one's body" has a greater influence on sexual drive than physical considerations alone.

A more general question is whether GSI reflects sexual condition or serves as an indirect modulator of SF through self-confidence. Are PFD questionnaires appropriate for addressing psychological factors like as shame and fear of judgment? Furthermore, the effect of altering GSI via psychotherapy or sexual health education on long-term sexual quality of life should be studied. Future research should concentrate on multifactorial models and samples representing a broader range of cultures and ages.
