# Supplementary material for: Comments on: "Relationship involving sexual function, distress symptoms of pelvic floor dysfunction, and female genital self-image"
Source: Rev Bras Ginecol Obstet. 2025 Oct 21;47:e-rbgo83. doi: 10.61622/rbgo/2025rbgo83 (PMC12711238; doi:10.61622/rbgo/2025rbgo83)
Supplement: Related texts

# Author´s reply to comment on: "Relationship involving sexual function, distress symptoms of pelvic floor dysfunction, and female genital self-image"

**Authors:** Guilherme Tavares de Arruda, Gabrielle Peres Paines, Bianca Rangel da Silva, Lauren Xavier Pairé, Hedioneia Maria Foletto Pivetta, Melissa Medeiros Braz, Janeisa Franck Virtuoso

**Affiliations:** 1 Universidade Federal de São Carlos São Carlos SP Brazil Universidade Federal de São Carlos, São Carlos, SP, Brazil.; 2 Universidade Federal de Santa Maria Santa Maria RS Brazil Universidade Federal de Santa Maria, Santa Maria, RS, Brazil.; 3 Universidade Federal de Santa Catarina Araranguá SC Brazil Universidade Federal de Santa Catarina, Araranguá, SC, Brazil.

Dear Editor,

We would like to thank the authors for their thoughtful letter regarding our article, "Relationship Involving Sexual Function, Distress Symptoms of Pelvic Floor Dysfunction, and Female Genital Self-Image".^(1)^

We appreciate your thoughtful commentary regarding the noncausal effect of the analysis.^(2)^ As described in the Materials and Methods section, the study employed a cross-sectional and observational design, which does not allow for causal inference since temporal precedence between exposure and outcome cannot be assured.^(3)^ We identified this as one of the study's limitations. Additionally, multiple linear regression, as used in this study, is useful for exploring adjusted associations, but it cannot establish cause-and-effect relationships. Similarly, we carefully discuss the results regarding the R² values of the model. Although statistically significant, these values demonstrate low predictive capacity, suggesting the existence of other relevant factors not included in the model.

We agree with the authors that no controls or adjustments were made for important confounding factors. Our study did not control for important potential confounding variables such as menopausal status, chronic diseases (e.g., diabetes or hypertension), mental health conditions, or medication use (e.g., antidepressants). The manuscript itself acknowledges that neither depression diagnosis nor antidepressant use was investigated. Additionally, while the sample power was high (99%), only 114 of the 216 participants were included in the sexual function (SF) analysis because they had been sexually active in the past four weeks. The SF assessment requires this time frame for recall.^(4)^ As described in the Results, this substantial reduction in sample size may have introduced selection bias since excluding nearly half of the sample could result in a subgroup with different characteristics that influence the relationship between SF, pelvic floor dysfunction (PFD) symptoms, and genital self-image (GSI). Consequently, the generalizability of the findings to the broader female population is reduced.

The observed correlations between pelvic organ prolapse (POP) symptoms and GSI were weak (rho ≈ −0.28) (Table 2), even when they were statistically significant. This suggests that other unmeasured factors, such as self-esteem, cultural beliefs, and perceptions of attractiveness, may play a substantial role. Conceptually, GSI can act as both a reflection of sexual health and an indirect modulator of SF via self-confidence and self-esteem.^(5)^ The complexity of this relationship calls for broader analyses, such as mediation models, to help identify indirect relationships between variables. As the authors stated, future studies could examine the impact of psychotherapeutic or educational interventions on GSI and their subsequent effects on SF.

As mentioned in the letter, the criticisms in the letter to the editor are supported by the limitations we explicitly acknowledged in the manuscript and by established methodological principles. While our study provides valuable insights into the relationship between SF, PFD symptoms, and GSI, future research should employ longitudinal designs, utilize more representative samples, adjust for confounding variables, and include psychosocial factors. These improvements will enhance our understanding of causal pathways and the clinical applicability of the findings.

We are once again grateful for the authors’ contribution to this important discussion, and we hope that our response has adequately addressed their concerns.

## References

[1] Arruda GT, Paines GP, Silva BR, Pairé LX, Pivetta HM, Braz MM (2023). Relationship involving sexual function, distress symptoms of pelvic floor dysfunction, and female genital self-image. Rev Bras Ginecol Obstet.

[2] Daungsupawong H, Wiwanitkit V (2025). Comments on: "Relationship involving sexual function, distress symptoms of pelvic floor dysfunction, and female genital self-image". Rev Bras Ginecol Obstet.

[3] Pearce N (2012). Classification of epidemiological study designs. Int J Epidemiol.

[4] Neijenhuijs KI, Hooghiemstra N, Holtmaat K, Aaronson NK, Groenvold M, Holzner B (2019). The Female Sexual Function Index (FSFI)-a systematic review of measurement properties. J Sex Med.

[5] Silva ML, Moraes RS, Prada MB, Silva Júnior M, Cunha KC, Jácomo R (2024). Association of body image and genital self-image with female sexual function: a systematic review. J Sex Marital Ther.
